# Supplementary material for: A 3D printed plastic frame deeply impacts yeast cell growth
Source: Front Bioeng Biotechnol. 2023 Sep 12;11:1250667. doi: 10.3389/fbioe.2023.1250667 (PMC10523559; doi:10.3389/fbioe.2023.1250667)
Supplement: Supplementary file 2 [file DataSheet1.docx]

A 3D printed plastic frame deeply impacts yeast cell growth

Esther Molina-Menor^1^, Àngela Vidal-Verdú^1^, Carlos Gomis-Olcina^1^, Juli Peretó^1,2^ and Manuel Porcar^1,3*^

^1^ Institute for Integrative Systems Biology (I2SysBio), Universitat de València-CSIC, Paterna, Spain

^2^ Departament de Bioquímica i Biologia Molecular, Universitat de València, Spain

^3^ Darwin Bioprospecting Excellence SL, Parc Científic Universitat de València, Spain

*** Correspondence:**Corresponding Author
[manuel.porcar@uv.es](mailto:manuel.porcar@uv.es)

# Differential protein expression analysis: SWATH

*Sample preparation*

Cell pellets were thawed, resuspended in 250 μL of 2X Laemmli lysis buffer pH 6.8 (from 4X Laemmli: 277.8 mM Tris-HCl, 44.4 % (v/v) glycerol, 4.4 % LDS, 0.02% bromophenol blue, 10 % β-mercaptoehanol) and transferred to 1.5 mL FastPrep tubes with 250 μL of glass beads. Cells were disrupted in a FastPrep homogenizer (45 s full-speed cycles separated by 5 min on ice), centrifuged for 15 in at 20000 g and supernatants (protein extracts) were finally transferred to clean tubes.

*Spectral library building*

The spectral library was constructed by mixing equivalent aliquots of all the samples (100 μg). Mixed protein extracts were charged and separated by 1D SDS PAGE electrophoresis. The career of the library was divided into 4 pieces and, the samples were digested with 500 ng sequencing grade trypsin (Promega) (Shevchenko et al., 1996) at 37 °C. The digestion was stopped with 10 % trifluoroacetic acid (TFA). The supernatant (SN) was removed and library gel slides dehydrated with pure acetonitrile (ACN). The new peptide solutions were combined with the corresponding SN. The peptide mixtures were dried in a speed vacuum and resuspended in 2 % ACN; 0.1 % TFA (40μL).

*LC-MSMS Analysis for library*

The proteins were identified through LC-MSMS (Ekspert nanoLC 425, Eksigent; nanoESI qQTOF 6600plus Triple TOF, Ab Sciex). The peptide mixture sample (5 μl) was loaded onto a trap column (3μ C18‐CL 120 Ᾰ, 350 μ m x 0.5mm; Eksigent) and desalted with 0.1% TFA at 5 μl/min for 3 min. Then, peptides were loaded onto an analytical column (3μ C18‐CL 120 Ᾰ, 0.075 x 150 mm; Eksigent), equilibrated in 5% ACN- 0.1% formic acid (FA) and eluted with a linear gradient of 7-40 % B in A for 60 min (A: 0.1 % FA; B: ACN, 0.1 % FA) at a flow rate of 300 nL/min.

Eluted peptides were ionized at 2.8 kV. Data-dependent mode was used for analysis (DDA). Survey MS1 scans were acquired from 350–1250 m/z for 250 ms. The quadrupole resolution was set to ‘UNIT’ for MS2 experiments. Data were acquired 100–1500 m/z for 150 ms in ‘high sensitivity’ mode. Up to 25 were selected for fragmentation after each scan. Dynamic exclusion was set to 15 s. Rolling collision energies equations were set according to the following equation: |CE|=(slope)x(m/z)+(intercept).

*Protein identification*

ProteinPilot (v5.0, Ab Sciex) was used for protein identification with default parameters. The Paragon algorithm was used to search the Uniprot-*Saccharomyces* database. Protein grouping was done by Pro group algorithm.

*SWATH analysis of individual samples*

Differential proteomic analysis was carried out through SWATH, by loading 20 μg of total protein extract in a 1D SDS PAGE gel. Gel fractions were then cut and digested with 500 ng of sequencing grade trypsin (Promega) in 150 μl of ABC solution, as described by Svechenko et al. (1996). The digestion was stopped with TFA at a final concentration of 1%, followed by a double extraction with ACN. Samples were then dried in a rotatory evaporator and finally resuspended in 40 μl 2 % ACN-0.1 % TFA.

*SWATH LC-MSMS Analysis*

5 μl of digested bands proteins were loaded onto a trap column (LC Column, 12 nm, 3 μ Triart‐ C18, 0.5 x5.0 mm; YMC) and desalted with 0.1% TFA at 10 μl/min during 5 min. The peptides were then loaded onto an analytical column (LC Column, 3 μm ChromoXP C18, 150 x 0.3 mm, Capillary Eksigent) equilibrated in 3% acetonitrile 0.1% FA (formic acid). Elution was carried out with a linear gradient of 8a35% B in A for 60 min. (A: 0.1% FA; B: ACN, 0.1% FA) at a flow rate of 5 μL/min. Peptides were analyzed in a mass spectrometer microESI qQTOF (6600plus TripleTOF, ABSCIEX). Sample was ionized in a Source Type: Optiflow 1‐50 μL Micro applying 4.5 kV to the spray emitter. Survey MS1 scans were acquired from 400–1250 m/z for 250 ms. The tripleTOF was operated in swath mode. 100 variable windows from 400 to 1250 m/z were acquired throughout the experiment. The total cycle time was 2.79 sec. The quadrupole resolution was set to ‘UNIT’ for MS2 experiments. Data were acquired 100–1500 m/z for 25 ms in ‘high sensitivity’ mode.

*Data analysis and protein quantification*

Peak View 2.1 was used for protein quantification with the following parameters: 20 peptides per protein, 6 transitions per protein, 95 % peptide confidence threshold, 1 % false discovery rate, 5 min XIC Extraction window and 25 ppm XIC width. Retention times were calculated using iRT calibration peptides. With this, 1180 proteins were quantified in the 9 samples.

Marker View (Sciex) was used to analyze the data. Protein areas were calculated by normalizing the total protein areas. Statistical test of reduction of dimensionality, specifically Principal Component Analysis (PCA, Pareto scaling) was carried out. T-test of differential expression was applied between pairs of conditions.

**References**

Shevchenko, A., Jensen, O. N., Podtelejnikov, A. V., Sagliocco, F., Wilm, M., Vorm, O., Mortensen, P., Shevchenko, A., Boucherie, H., & Mann, M. (1996). Linking genome and proteome by mass spectrometry: large-scale identification of yeast proteins from two dimensional gels. *Proceedings of the National Academy of Sciences of the United States of America, 93*(25), 14440–14445. <https://doi.org/10.1073/pnas.93.25.14440>
